# Dialysis headache: characteristics, impact and cerebrovascular evaluation

**DOI:** 10.1590/0004-282X-ANP-2021-0133

**Published:** 2022-03-13

**Authors:** Eduardo SOUSA MELO, Rodrigo Pinto PEDROSA, Filipe CARRILHO AGUIAR, Lucila Maria VALENTE, Pedro Augusto SAMPAIO ROCHA-FILHO

**Affiliations:** 1 Universidade Federal de Pernambuco, Programa de Pós-Graduação em Neuropsiquiatria e Ciências do Comportamento, Recife PE, Brazil. Universidade Federal de Pernambuco Programa de Pós-Graduação em Neuropsiquiatria e Ciências do Comportamento Recife PE Brazil; 2 Universidade de Pernambuco, Pronto Socorro Cardiológico de Pernambuco, Recife PE, Brazil. Universidade de Pernambuco Pronto Socorro Cardiológico de Pernambuco Recife PE Brazil; 3 Clínica Multirim, Recife PE, Brazil. Clínica Multirim Recife PE Brazil; 4 Universidade Federal de Pernambuco, Centro de Ciências Médicas, Divisão de Nefrologia, Recife PE, Brazil. Universidade Federal de Pernambuco Centro de Ciências Médicas Divisão de Nefrologia Recife PE Brazil; 5 Universidade Federal de Pernambuco, Divisão de Neuropsiquiatria, Recife PE, Brazil. Universidade Federal de Pernambuco Divisão de Neuropsiquiatria Recife PE Brazil; 6 Universidade de Pernambuco, Hospital Universitário Oswaldo Cruz, Recife PE, Brazil. Universidade de Pernambuco Hospital Universitário Oswaldo Cruz Recife PE Brazil

**Keywords:** Headache, Pain, Renal Dialysis, Anxiety, Quality of Life, Ultrasonography, Doppler, Transcranial, Cefaleia, Dor, Diálise Renal, Ansiedade, Qualidade de Vida, Ultrassonografia Doppler Transcraniana

## Abstract

**Background:**

Headache is one of the most frequent symptoms that occur during hemodialysis sessions. Despite the high prevalence of dialysis headache, it has been little studied.

**Objective:**

To evaluate the characteristics, impact and factors associated with dialysis headache. The behavior of the cerebral vasculature was also compared between patients with and without dialysis headache.

**Methods:**

This was a cross-sectional study. Consecutive patients who underwent hemodialysis were assessed through a semi-structured questionnaire, the Headache Impact Test (HIT-6), the Hospital Anxiety and Depression Scale and the Short Form-36 Health Survey (SF-36). Transcranial Doppler ultrasonography was performed in the first and fourth hours of hemodialysis.

**Results:**

A total of 100 patients were included; 49 of them had dialysis headache. Women (OR=5.04; 95%CI 1.95-13.04), younger individuals (OR=1.05; 95%CI 1.01-1.08), individuals with higher schooling levels (OR=3.86; 95%CI 1.4-10.7) and individuals who had spent longer times on dialysis programs (OR=0.99; 95%CI 0.98-1) had more dialysis headache (logistic regression). Individuals with dialysis headache had worse quality of life in the domains of pain and general state of health (56.9 *versus* 76.4, p=0.01; 49.7 *versus* 60.2, p=0.03, respectively). Dialysis headache was associated with significantly greater impact on life (OR=24.4; 95%CI 2.6-226.6; logistic regression). The pulsatility index (transcranial Doppler ultrasonography) was lower among patients with dialysis headache than among those without them.

**Conclusions:**

Dialysis headaches occur frequently and are associated with worse quality of life and patterns of cerebral vasodilatation.

## RESUMO

**Antecedentes:**

A cefaleia é um dos sintomas mais frequentes que ocorrem durante as sessões de hemodiálise. Apesar da alta prevalência, essa cefaleia é pouco estudada.

**Objetivo:**

Avaliar as características, impacto e fatores associados à cefaleia da diálise. O comportamento da vasculatura cerebral também foi comparado entre pacientes com e sem cefaleia da diálise.

**Métodos:**

Este foi um estudo transversal. Pacientes consecutivos submetidos à hemodiálise foram avaliados por meio de questionário semiestruturado, do Headache Impact Test (HIT-6), Hospital Anxiety and Depression Scale e Short Form-36 Health Survey (SF-36). Foi realizada ultrassonografia Doppler transcraniana na primeira e na quarta horas de hemodiálise.

**Resultados:**

Foram incluídos 100 pacientes, 49 deles tinham cefaleia da diálise. Mulheres (OR=5,04; IC95% 1,95-13,04), indivíduos mais jovens (OR=1,05; IC95% 1,01-1,08), com maior escolaridade (OR=3,86; IC95% 1,4-10,7) e que passaram mais tempo em programas de diálise (OR=0,99, IC95% 0,98-1) tiveram mais cefaleia da diálise (regressão logística). Indivíduos com cefaleia dialítica tiveram pior qualidade de vida nos domínios dor e estado geral de saúde (56,9 *versus* 76,4, p=0,01; 49,7 *versus* 60,2, p=0,03, respectivamente). A cefaleia da diálise foi associada a um impacto significativamente maior na vida (OR=24,4; IC95% 2,6-226,6; regressão logística). O índice de pulsatilidade (ultrassonografia Doppler transcraniana) foi menor entre os pacientes com cefaleia da diálise do que entre aqueles sem.

**Conclusões:**

A cefaleia da diálise ocorre com frequência e está associada a pior qualidade de vida e a padrões de vasodilatação cerebral.

## INTRODUCTION

Headache is one of the most frequent symptoms that occur during hemodialysis sessions. Around 70% of hemodialysis patients present headaches^1^. It has been estimated that 28 to 73% of hemodialysis patients can be considered to be suffering from dialysis headache^2^^,^^3^^,^^4^.

The physiopathology of dialysis headache remains unclear. Factors that are known to be associated with these headaches include the type of dialysis solution used (acetate presents greater risk of dialysis headache than bicarbonate)^5^; variations in urea, sodium and magnesium levels and in arterial blood pressure^2^^,^^3^^,^^6^; levels of calcitonin gene-related peptide (CGRP) and levels of substance P during dialysis^7^. The blood-brain barrier may have an important role in the appearance of this headache. The concentration gradient between the brain and the blood that occurs during dialysis, with consequent passage of free water through the blood-brain barrier may lead to cerebral edema in some patients, thus consequently causing headache^3^^,^^8^.

Despite the high prevalence of dialysis headache, it has been little studied. This leads to difficulty in recognizing its characteristics, understanding its physiopathological mechanisms and knowing how to manage it. The aim of the present study was to evaluate the frequency, characteristics and impact of these headaches and the factors associated with them. The behavior of the cerebral vasculature, as assessed using transcranial Doppler ultrasonography, was also compared between patients with and without dialysis headache.

## METHODS

This was a cross-sectional study.

Patients with age 18 years and over, with chronic kidney failure attended through hemodialysis at either Hospital das Clínicas or the Clínica Multirim between September 2015 and January 2016 were included. In both of these services, bicarbonate was used in the dialytic solution and the duration of the hemodialysis sessions was four hours.

Patients were excluded in the following situations: use of hemodialysis therapy for less than six months; presence of cognitive impairment; altered consciousness or difficulty with verbal communication that caused difficulty in making assessments; presence of diseases that cause secondary headaches; or use of prophylactic medications against headaches.

This study was approved by the research ethics committee of the Health Sciences Center (CCS) of the Universidade Federal de Pernambuco (CAAE 47077715.3.0000.5208). All the patients who participated in this study signed a free and informed consent statement.

All the patients were evaluated by a neurologist with experience in diagnosing and treating headaches, who interviewed these patients and conducted clinical and neurological examinations before the hemodialysis sessions were started. Their headaches were classified in accordance with the diagnostic criteria of the beta version of the third edition of the International Classification of Headache Disorders (ICHD-3 beta)^9^ and were later reclassified in accordance with the criteria of the final third edition of the International Classification of Headache Disorders (ICHD-3)^10^. Patients were considered to be making excessive use of caffeine when they reported having six or more cups of coffee per day^11^.

Patients were considered to present dialysis headache if they fulfilled the criterion of having had at least three episodes of acute headache with two of the following situations of causality: 1 -the headache needed to have started during the hemodialysis session; 2 -the headache needed to have worsened during the hemodialysis session and/or to have resolved within 72 hours after the end of the session^10^.

The following questionnaires were applied before the hemodialysis sessions:

- A semi-structured questionnaire that sought the following: sociodemographic data; data on the chronic kidney failure and its treatment (etiology, time when hemodialysis was started and medications used); data on the presence and characteristics of primary headaches (time when they started; duration of the attacks; frequency; pattern of pain; factors associated; and medications used); and data on the presence and characteristics of dialysis headache (the very first occurrence of dialysis headache, in relation to the start of the dialysis treatment; time at which pain generally started, in relation to the start of the hemodialysis session; duration of the attack; frequency; pattern of pain; factors associated; factors that improved or worsened the pain; and medications used).
- Headache Impact Test (HIT-6): used to estimate the impact of headaches on the patient’s life^12^. The higher the score is, the greater the impact of the headache is. Scores greater than or equal to 60 are considered to have “severe impact”; scores between 56 and 59 points, “substantial impact”; scores between 50 and 55 points, “some impact”; and scores less than or equal to 49, “minimal or no impact”^13^. Patients who did not present headaches were classified in the category “minimal or no impact”.
- Hospital Anxiety and Depression Scale (HADS): this allows the presence of symptoms of anxiety and depression to be diagnosed. It is subdivided into two subscales: one for anxiety and the other for depression^14^^,^^15^. Presence of anxiety and depression is defined as a score ≥8 on the respective subscale^16^.
- Short Form-36 Health Survey (SF-36): this enables evaluation of health-related quality of life in eight domains: physical functioning, role-physical, pain, general state of health, vitality, social functioning, role-emotional and mental health. The lower the score is, the worse the quality of life is^17^.

### Evaluation via transcranial Doppler ultrasonography

Transcranial Doppler ultrasonography was performed using the DWL-EZbox^®^ device, by means of transtemporal windows. The middle cerebral arteries were evaluated bilaterally, at depths of between 40 and 60 mm, every 2 mm, with regard to the parameters of mean flow velocity (cm/sec) and pulsatility index (mean vascular resistance). The latter was calculated as the ratio of the difference between systolic and diastolic velocities divided by the mean velocity. This examination was performed in the first and fourth hours of hemodialysis, by the same observer, with the patient either sitting or lying down.

### Statistical analysis

The Statistical Package for the Social Sciences for Windows package, version 21.0 (SPSS Inc., IBM Company, Chicago, IL, USA), was used for the statistical analysis.

The descriptive analysis included calculation of means (with standard deviation) or medians (with interquartile range) for the continuous variables, and absolute distributions (with percentage) for the categorical variables.

The Kolmogorov-Smirnov test was used to assess whether the data presented normal distribution. The numerical variables were compared between groups using the t-test if the distribution was normal; or using the Mann-Whitney test if the distribution was non-normal. The percentage distributions of the categorical variables were compared between the groups using the chi-square test or Fisher’s exact test.

All the statistical tests were two-tailed and the significance level taken was based on α of 0.05.

Logistic regression was performed to determine the predictors for dialysis headache. Variables with p<0.15 in the univariate analysis were included in the model using a stepwise method, and those with p<0.1 were kept in the model. The following variables were evaluated at this stage: sex, age, schooling level, length of time for which the patient had been undergoing hemodialysis, presence of anxiety and previous migraine.

Another logistic regression was then performed to determine the factors associated with the functional impact of the headache, as evaluated using the HIT-6 scale. Variables presenting p<0.1 in the univariate analysis were included in the model using a stepwise method and those with p<0.1 were kept in the model. The following variables were evaluated at this stage: sex, age, schooling level, family income, presence of anxiety, presence of depression, previous tension-type headache, previous migraine and presence of dialysis headache.

### Availability of data and materials

The datasets used and/or analyzed during the current study are available from the corresponding author upon reasonable request.

## RESULTS

A total of 100 patients were evaluated and included in this study, among whom 52 were undergoing dialysis at Hospital das Clínicas and 48 at the Clínica Multirim. Their mean age was 51.8 years (±13.6); 50% were women; 53% were married; their mean schooling level was 7.7 years (±3.5); and 41% had a family income of up to two Brazilian minimum monthly wages (one monthly minimum wage was equivalent to approximately US$ 200.00).

These patients had been undergoing hemodialysis for a mean period of 54 months (±59.3) and had had their diagnosis of chronic kidney disease (CKD) for a mean period of 100.4 months (±113.3).

The main etiologies for CKD were hypertensive nephropathy (39%), diabetic nephropathy (16%), glomerulopathy (10%) and chronic tubulointerstitial nephritis (10%). A variety of causes were presented by the remaining 25%: polycystic kidney disease, tuberculosis, nephrectomy due to trauma, nephrectomy due to kidney cancer, hemolytic-uremic syndrome, scleroderma and Takayasu’s arteritis.

There were 76 patients with primary headaches: 25 with migraine and 51 with tension-type headache. Anxiety was present in 28% and depression in 25%.

Forty-nine patients had headaches that fulfilled the criteria for dialysis headache. Among these patients, it had a severe impact on 16 (32.7%); a substantial impact on 4 (8.2%); some impact on 7 (14.3%); and minimal or no impact on 22 (44.9%).

Table 1 shows the characteristics of the dialysis headache. The headache pattern that was most often found was tension-type headache. In most cases, the headache started insidiously in the third or fourth hour of hemodialysis. Its intensity and frequency became lower than at the onset of the condition, over a period of months.

**Table 1.** Characteristics of the dialysis headache (n=49).

Characteristic		n (%)	mean (±SD)
Start	Insidious	39 (79.6%)	
	Sudden	10 (20.4%)	
Characteristic of headache	Throbbing	36 (73.5%)	
	Pressing	11 (22.4%)	
	Stabbing	2 (4.1%)	
Intensity (VAS)			6.7 (±2.1)
Duration (minutes)			215.2 (±429.2)
Photophobia		18 (36.7%)	
Phonophobia		21 (42.9%)	
Worsening through exercise		9 (18*.*4%)	
Nausea		24 (49%)	
Vomiting		14 (28.6%)	
Aura		3 (6.1%)	
Autonomic signs		1 (2%)	
Start of headache in relation to dialysis (hour)	First	1 (2%)	
	Second	8 (16.3%)	
	Third	16 (32.7%)	
	Fourth	24 (49%)	
Location	Bilateral	34 (69.4%)	
	Unilateral	9 (18.3%)	
	Sometimes unilateral and sometimes bilateral	6 (12.2%)	
Headache pattern -n (%)	Tension-type	26 (53.1%)	
	Migraine	22 (44.9%)	
	Tension-type or migraine	1 (2%)	
Medication used during dialysis headache attack	Dipyrone	34 (69.4%)	
	Paracetamol	8 (16.3%)	
	Dipyrone/paracetamol	4 (8.2%)	
	Others	3 (6.1%)	
Dialysis headaches over the last 30 days		26 (53.1%)	
Number of sessions with headaches over the last 30 days			2 (±2.8)
Length of time until end of headache after end of dialysis (minutes)			180.4 (±421.1)
Behavior of headache intensity over the months	Same as in the beginning	12 (24.5%)	
	Becoming more intense	9 (18.4%)	
	Becoming less intense	28 (57.1%)	
Behavior of headache frequency over the months	Same as in the beginning	7 (14.3%)	
	Becoming more frequent	9 (18.4%)	
	Becoming less frequent	33 (67.3%)	

SD: standard deviation; VAS: visual analogue scale for pain (0-10).

Table 2 presents the patients’ characteristics in relation to dialysis headache. Women, younger individuals, individuals with higher schooling levels and individuals who had been on hemodialysis programs for longer times presented dialysis headache significantly more frequently (shown through logistic regression).

**Table 2.** Associations of sociodemographic and clinical characteristics, compared between groups with and without dialysis headache.

Characteristics		With dialysis headaches	Without dialysis headaches	p-value	***Odds Ratio* (95%CI)**	p-value
		n=49	n=51	(Univariate analysis)	(Logistic regression)	(Logistic regression)
Age (years): median (IQR)		45 (39-58.9)	56 (49.5-64)	<0.01	1.05 (1.01-1.08)	<0.01
Sex	Female	34 (69.4%)	16 (31.4%)	<0.01	5.04 (1.95-13.04)	<0.01
	Male	15 (30.6%)	35 (68.6%)		Reference value	
Schooling level	Up to 7 years	11 (22.5%)	26 (51%)	0.07	Reference value	0.02
	≥8 years	38 (77.5%)	25 (49%)		3.86 (1.4-10.7)	
Length of time on hemodialysis (months): median (IQR)		36 (17-96)	24 (11-48)	0.13	0.99 (0.98-1)	0.04
Migraine		17 (34.7%)	8 (15.7%)	0.03	-	-
Tension-type headache		22 (44.9%)	29 (56.9%)	0.23	-	-
Excessive use of caffeine		3 (6.1%)	4 (7.8%)	0.73	-	-
Anxiety		18 (36.7%)	10 (19.6%)	0.06	-	-
Depression		14 (28.6%)	11 (21.6%)	0.42	-	-

IQR: interquartile range; 95%CI: 95% confidence interval.

**Figure 1.**
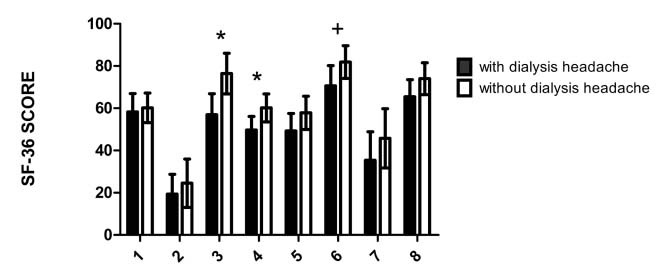
Quality of life and dialysis headache.

Figure 1 compares quality of life between individuals with and without dialysis headache. Those with dialysis headache presented significantly worse quality of life in the domains of pain and general state of health, in the SF-36 questionnaire.

Mean±SD; *p<0.05; +p=0.06; 1: physical functioning; 2: role-physical; 3: pain; 4: general state of health; 5: vitality; 6: social functioning; 7: role-emotional; 8: mental health.

Table 3 presents the associations between the patients’ characteristics and the impact of their headaches. Presenting anxiety and having dialysis headache were significantly associated with greater impact from headaches (shown through logistic regression).

**Table 3.** Associations of sociodemographic and clinical characteristics with the impacts from headaches.

Characteristics		High impact from headache	Low impact from headache	p-value	***Odds Ratio* (95%CI)**	p-value
		(HIT-6>55)	(HIT-6<56)	(Univariate analysis)	(Logistic regression)	(Logistic regression)
		n=21	n=79			
Age (years): median (IQR)		45 (38-53.5)	55 (44.5-61.5)	0.07	-	-
Sex	Female	18 (85.7%)	32 (40.5%)	<0.01	-	-
	Male	3 (14.3%)	47 (59.5%)			
Length of time on hemodialysis (months): median (IQR)		35 (20.5-78)	27 (12-78)	0.49	-	-
Migraine		11 (52.4%)	14 (17.7%)	0.02	-	-
Tension-type headache		7 (33.3%)	44 (55.7%)	0.07	-	-
Excessive use of caffeine		2 (9.5%)	5 (6.3%)	0.61	-	-
Anxiety		13 (61.9%)	15 (19%)	<0.01	8.8 (1.9-40.8)	<0.01
Depression		9 (42.9%)	16 (20.3%)	0.04		
Dialysis headache		20 (95.2%)	29 (36.7%)	<0.01	24.4 (2.6-226.6)	<0.05

IQR: interquartile range; HIT-6: Headache Impact Test.

Among the 100 patients evaluated, 83 underwent transcranial Doppler ultrasonography. The other 17 patients did not present a transtemporal bone window that was adequate for performing the examination. Table 4 shows a comparison of transcranial Doppler measurements between patients with and without the diagnosis of dialysis headache. The pulsatility index, which evaluates the resistance of the vessels studied, was significantly lower in the group with dialysis headache, in the right and left middle cerebral arteries in the first hour of hemodialysis and in the left middle cerebral artery in the fourth hour of dialysis. The mean flow velocities did not present any statistically significant differences.

**Table 4.** Comparison of Doppler measurements between patients with and without dialysis headache.

	With dialysis headache	Without dialysis headache	p-value
	(Mean ±SD)	(Mean ±SD)	
MFV of right MCA in 1^st^ hour of dialysis (n=83)	51.30 (±14.2)	46 (±12.2)	0.13
MFV of left MCA in 1^st^ hour of dialysis (n=83)	51.40 (±16.3)	47 (±12.8)	0.38
MFV of right MCA in 4^th^ hour of dialysis (n=83)	45.60 (±16.8)	41.4 (±10.9)	0.55
MFV of left MCA in 4^th^ hour of dialysis (n=83)	43.20 (±15.2)	41.9 (±13.4)	0.98
PI of right MCA in 1^st^ hour of dialysis (n=83)	0.97 (±0.25)	1.1 (±0.26)	0.01
PI of left MCA in 1^st^ hour of dialysis (n=83)	0.99 (±0.31)	1.09 (±0.25)	0.02
PI of right MCA in 4^th^ hour of dialysis (n=83)	1.08 (±0.3)	1.2 (±0.42)	0.13
PI of left MCA in 4^th^ hour of dialysis (n=83)	1.04 (±0.33)	1.21 (±0.41)	0.02

MFV: mean flow velocity; MCA: middle cerebral artery; PI: pulsatility index.

## DISCUSSION

In our study, the frequency of dialysis headache was 49%. In other studies, the prevalence of dialysis headache has ranged from 28 to 73%^2^^,^^3^^,^^4^^,^^5^^,^^6^. These other studies used different criteria for making the diagnosis of dialysis headache, which makes comparisons difficult. Our study was the first to use the ICHD-3 criteria among hemodialysis patients.

We also found high prevalence of primary headaches among our hemodialysis patients. This was in line with the findings of previous studies^18^^,^^19^. However, there was no association between presentation of primary headaches and presentation of dialysis headache.

Most of our patients started to present their headaches after the second hour of dialysis. This was in line with previous reports in the literature^4^^,^^6^^,^^18^^,^^20^. The pattern of dialysis headache that was most frequently found resembled tension-type headache. This result diverges from what was found by Antoniazzi et al., who used the diagnostic criteria of ICHD-1 and found that the most frequent pattern was migraine^4^. Despite our finding of the predominance of the tension-type headache pattern, the migraine pattern was also frequently seen. The decrease in the severity of dialysis headache over time that our patients reported constitutes important prognostic information that needs to be confirmed through prospective studies.

As far as we are aware, this was the first study to evaluate the risk factors associated with dialysis headache. Women, younger individuals, individuals with higher schooling levels and individuals who had been on hemodialysis programs for longer times presented dialysis headache significantly more frequently. Higher prevalence of primary headaches such as migraine and tension-type headache among women and younger adults has previously been described^21^. We consider that the association between longer time spent on a hemodialysis program and development of dialysis headache is important clinical data for guiding these patients and for nephrologists. This finding may also have physiopathological significance. The conditions needed for developing this type of headache probably require a long time to become established.

Also as far as we know, this was the first study to assess the impact of dialysis headache. Among the headaches presented, dialysis headache was the only one that was significantly associated with a high negative impact on the patients’ lives. Although patients with kidney failure have other complications and other causes of pain, such as neuropathic pain and cramps^22^, presence of dialysis headache had a significant negative effect on our patients’ perceptions of their health. This pain interfered to a greater extent with their quality of life.

Cerebrovascular behavior among patients with dialysis headache had not previously been reported in the literature, to the best of our knowledge. The pulsatility index, which provides a measurement of vascular resistance, was significantly lower bilaterally in the middle cerebral arteries in the first hour of hemodialysis and in the left middle cerebral artery in the fourth hour of dialysis. This suggests to us that there was a pattern of cerebral dilatation in our patients with dialysis headache.

A previous study found that there was higher plasma concentration of calcitonin gene-related protein (CGRP) before hemodialysis in patients with dialysis headache, compared with controls^7^. CGRP is a potent vasodilator and our finding may corroborate the hypothesis that it participates in the physiopathology of dialysis headache. This molecule is widely distributed in the central and peripheral nervous systems and has an important role in mechanisms favoring inflammation, nociception and hyperalgesia^23^^,^^24^.

Antoniazzi and Corrado suggested that nitric oxide (NO) might participate in the physiopathology of dialysis headache^19^. NO is a vasodilator and the increase in its levels over the course of dialysis might provide an explanation for why headaches generally occur after the second hour of dialysis. This might also explain the vasodilatation pattern that we found at the end of the dialysis among the patients with dialysis headache. This state of vasodilatation among these patients may suggest that compensatory cerebral self-regulation mechanisms had failed, as previously seen among patients with intracranial atherosclerotic disease, which led to loss of vasoreactivity^25^.

Our study presents some limitations. Our sample was selected according to convenience and may not have represented the population of patients who undergo hemodialysis. We did not measure the hematocrit levels of our patients. These might become altered during hemodialysis and such occurrences would be related to vascular resistance and oxygen transportation capacity, which could lead to a metabolically mediated pattern of vasodilatation or vasoconstriction^26^.

The evaluation using transcranial Doppler ultrasonography was done only on the middle cerebral arteries. However, cerebrovascular phenomena may occur asymmetrically and, depending on the patient’s clinical condition, these may occur more in the vertebrobasilar region. This, and the fact that few of our patients presented headaches at the time of our evaluation, may have given rise to underestimation of the vascular alterations. Such alterations might thus have been greater than what we found.

In conclusion, dialysis headaches have high frequency and generally start after the second hour of dialysis, and their pattern most frequently consists of tension-type headache. Dialysis headache has a significant negative impact on quality of life and occurs more frequently among women, younger adults, individuals with higher schooling levels and individuals who have been on hemodialysis programs for longer times. Patients with a diagnosis of dialysis headache present a pattern of cerebral vasodilatation.
